# Cervical Cancer Screening Acceptance among Women in Dabat District, Northwest Ethiopia, 2017: An Institution-Based Cross-Sectional Study

**DOI:** 10.1155/2020/2805936

**Published:** 2020-02-07

**Authors:** Meried Eshete, Mohammedbirhan Abdulwuhab Atta, Hedija Yenus Yeshita

**Affiliations:** ^1^Government Public Health Facility, Gondar, Ethiopia; ^2^Department of Pharmacology, School of Pharmacy, University of Gondar, Gondar, Ethiopia; ^3^Institute of Public Health, Department of Reproductive Health, University of Gondar, Gondar, Ethiopia

## Abstract

**Background:**

Cervical cancer is a global health problem. It is the second most common cancer in women worldwide, and it is the most frequent form and the leading cause of cancer mortality among Ethiopian women. Cervical cancer screening can reduce at least 50% of cervical cancer deaths. In Ethiopia, practice of cervical cancer screening is below 1%. Hence, this study aimed at assessing cervical cancer screening acceptance and determinant factors among women in Dabat district of Northwest Ethiopia.

**Methods:**

A community-based cross-sectional study design was conducted in Dabat district in Northwest Ethiopia, 2016. The multistage sampling method was used to recruit 790 women from the selected rural and urban kebeles. Data were collected using a structured questionnaire. Multivariate logistic regression analysis method was employed to determine factors significantly associated with the acceptance of cervical cancer screening with a 95% CI at *p* value <0.05.

**Results:**

The overall awareness of cervical cancer screening was 12.1% (95% CI: 9.6, 14.5), and 17.1% (95% CI 14.4, 19.8) of them accepted the screening. In multivariate logistic regression analysis, having knowledge about cervical cancer (AOR = 2.6, 95% CI: 1.7, 3.8), parity women who had more children (AOR = 3.1, 95% CI: 1.7, 5.5) and those who perceived the severity of the disease (AOR = 1.9, 95% CI (1.3–3.1)) were statistically significant factors for acceptance of cervical cancer screening.

**Conclusions:**

Most of the women had poor awareness and acceptance of cervical cancer screening. The findings also revealed that women of multiparous, knowledge about cervical cancer, and perceived the disease as severe were shown to be significant factors of acceptance for cervical cancer screening. Hence, continuous health education and appropriate counseling to women should be performed.

## 1. Background

Cervical cancer is a preventable noncommunicable disease of public health importance, and the second most common cancer in women. Worldwide, 80% of cervical cancer cases occurred in developing countries [1]. Global trends show that, in developing countries going through rapid societal and economic changes, the shift towards lifestyles like that of industrialized countries leads to a rising burden of cancers [2]. The incidence of cervical cancer varies greatly worldwide. There is a large difference between developing and developed countries, where cervical cancer cases have been significantly reduced since the implementation of effective screening programmes [3]. The success of any screening program will depend on proper rendering of services, health professionals, availability, low cost, and, above all, the awareness and attitude of women at the receiving end [4].

In Ethiopia, where ROCs are a widespread, but rare, acknowledged the problem [5]. Nearly out of 22 million Ethiopian women over the age of 15 years, approximately 7,600 are diagnosed with cervical cancer, and roughly 6,000 women die of the disease yearly [6]. Arranged screening is more cost-effective than opportunistic screening, making better use of available resources and ensuring that the greatest number of women will benefit [7].

Ethiopia has invested little in the infrastructure, training, and laboratory capacity required for successful pap smear screening [6]. Prevention and control are the most important public health strategies. Empowerment of women, education, earlier screening by affordable technology, and treatment of precancers are the most promising interventions to reduce the burden of cervical cancer [8].

### 1.1. Literature Review

#### 1.1.1. Cervical Cancer Screening Awareness

A study conducted in Indian urban women showed that 16.4% of women are aware of cervical cancer screening [9]. Similar study conducted in the southern part of the same country revealed that a majority of the women (81.9%) have poor knowledge about cervical cancer [10]. A study conducted in Nigeria on HIV-positive women showed that 56.2% and 34.5% respondents were aware on cervical cancer disease and screening/test, respectively [11]. In the southeastern part of the same country, less than 37.5% of the women were aware on cervical cancer; about 30% knew that it was preventable; 25% were aware of cervical screening, but 20% knew the screening center [12]. In Gabonese women, only 27.9% had heard about cervical cancer screening [13].

Regarding knowledge about cervical cancer screening and perception of risks among women attending outpatient clinic in rural Kenya, out of 419 participants, 327 (78.0%) had heard of cervical cancer screening, 288 (68.7%) women felt at risk for cervical cancer, and 333 (79.5%) stated that they would undergo screening if offered [14].

Facility-based study in Ethiopia, Addis Ababa, among women living with HIV indicated that 97 (88.2%) participants believed as the disease is preventable, and 31.4% knew the availability of the screening procedures for the disease [15]. In the same city of the country, among reproductive health service clients, 478 (91.9%) and 222 (42.7%) women heard about cervical cancer and cervical cancer screening, respectively [16]. In a similar study conducted in Ethiopia, about 71% of participants had ever heard of cervical cancer. Among women who had ever heard of cervical cancer, 49% did not know the cause, while 74% were able to identify at least one risk factor for cervical cancer [17].

#### 1.1.2. Cervical Cancer Screening Acceptance

A hospital-based study in Nigeria showed that 62.5% of respondents have the willingness to be screened for cervical cancer [12]. In a study conducted in the same country, among HIV-positive women, 79.8% respondents accepted to take cervical cancer screening [11]. A study in Uganda indicates that 63% of women reported intention to screen for cervical cancer [18]. In a similar study in Burkina Faso, 96.67% would accept to be screened, and 11.07% were screened for cervical cancer [19]. In a study in Addis Ababa from reproductive clients, 132 (37.9%) of participants strongly agreed that cervical cancer screening prevents cervical cancer, and 158 (45.4%) of women were willing to undergo cervical cancer screening [16].

In a facility-based study in the same city among women living with HIV, 62.7% was willing to screen for cervical cancer, and a quarter (24.8%) of them decided to be screened in the near future [15].

#### 1.1.3. Factors Associated with Cervical Cancer Screening Acceptance

A study among HIV-positive Nigerian women showed that cost of the test and religious denial were the most common reasons given for refusal to take the test [11]. Women aged forty-five and greater, having above 12-grade educational level, awareness of cervical cancer and screening services, believed that cervical cancer could be prevented, and believed screening could improve survival were more likely to access cervical screening [15, 20–22].

Females who had prior knowledge of cervical cancer screening, the source of information from health professionals, perceived the threat from cervical cancer or seriousness of the disease, and monthly income were associated with cervical cancer screening acceptance [15, 16, 20–22].

Awareness of the magnitude of the disease and its preventive measures among women are important to all efforts to reduce the burden of cervical cancer. Hence, the community-based survey was carried out to assess women's acceptability regarding cervical cancer screening and associated factors among women in Dabat district, Northwest Ethiopia. This study may help policymakers to decentralize cervical cancer screening services at the grass root levels (at the health centers) and may help the health workers to incorporate cervical cancer screening services and education within their activities.

## 2. Methods

### 2.1. Study Design and Period

A community-based cross-sectional study design was employed from January 2017 to March 2017 in Dabat district, Northwest Ethiopia.

### 2.2. Study Area

The study was conducted in Dabat district, Northwest Ethiopia, which is located 75 km north of Gondar town. The district has a total population of 145,458 who resides in thirty kebeles including urban and rural [23]. It also has 41 functioning governmental health institutions, out of which six of them are health centers and 35 health posts (31 rural and 4 urban) that provide curative and preventive services, but none of them has cervical cancer screening program [24].

### 2.3. Source and Study Population

The source population for this study was all women 18 years of age and above living in Dabat district and in the selected kebeles for the study. All women aged 18 years and above who lived in the selected kebeles were included in the study. Those women who were seriously ill and unable to communicate due to their illness were excluded from the study.

### 2.4. Sample Size Determination and Sampling Procedure

The sample size was calculated using a single proportion formula. The proportion of cervical cancer screening acceptance was 42.7% [19], 95% confidence interval, margin of error taken as 0.05, design effect 2, and adding nonresponse rate of 5%; the final sample size was 790.

### 2.5. Sampling Methods and Procedures

The multistage random sampling method was employed. The district of Dabat has a total of 30 kebeles of which 26 are rural and four of them are urban. Five kebeles from rural and one kebele from the urban were selected by using simple random sampling method (lottery method). The household was selected by using a systematic random sampling method, and women aged 18 years and above in the selected house were interviewed. But, if there are no eligible women in the selected household, the women in the next household were interviewed.

### 2.6. Data Collection Procedure and Tools

Pretested structured interviewer-administered questionnaire was used to collect the data. The questionnaire was first prepared in English and translated into Amharic. Variables on sociodemographic characteristics, knowledge, awareness, acceptance, and related factors for cervical cancer screening were included in the questionnaire. Six health extension workers and two supervisors were recruited from each kebele for data collection. The questionnaire was developed using questions from previously published articles [11, 15, 22].

### 2.7. Variables of the Study

#### 2.7.1. Dependent and Independent Variables

Acceptance of cervical cancer screening was the dependent variable. The independent variables, age, educational status, occupation, religion, residence, family income, and parity, were sociodemographic variables. Facility-related factors were availability and accessibility of cervical cancer screening and availability of trained health personnel. Personal factors were knowledge, perceived susceptibility, and severity of the disease (cervical cancer).

#### 2.7.2. Operational Definitions

*(1) Knowledgeable*. it refers to the knowledge of women regarding cervical cancer in general; in this study, if participants at least heard about the term cervical cancer, knowing at least a one risk factor and a symptom for cervical cancer, were referred to as knowledgeable [10, 17].

*(2) Perceived Severity of the Disease*. Perceptions are feelings concerning the seriousness of contracting cervical cancer or of leaving it untreated and measured by questions: is cervical cancer a deadly disease? and how severe is cervical cancer? [20].

*(3) Awareness of Cervical Cancer Screening*. It refers to participants at least having an affirmative answers to the questions: “have you ever heard about cervical cancer screening/PAP test?” “aware about the availability of cervical cancer screening?” and knowing the institution “where the screening is done?” [11, 13].

*(4) Acceptance of Cervical Cancer Screening*. It refers to the screening for cervical cancer if a woman is willing for testing and deciding to be screened in the near future. In this study, acceptance is evaluated by willingness (voluntariness) for screening and decides to be screened within the next two months [15].

### 2.8. Data Management and Quality Control

Data collectors and supervisors were given for two days of training on client approach mechanism, ethical issues, data collection instrument, and objective of the study. The supervisors have done a routine checkup of the questionnaire for completeness and consistency of the data. The tool was pretested before the actual data collection to check for the accuracy of responses, language clarity, and appropriateness of the tool.

### 2.9. Data Processing and Analysis

The questionnaire was checked visually, coded, and entered into Epi-info and transported to SPSS version 20 software package for analysis. Descriptive statistics such as numerical summary measures or frequencies were used for describing the study population in relation to relevant variables. Binary logistic regression analysis with a 95% confidence interval was used to assess the degree of association between dependent and independent variables and significance of the association. Variables which had a significant association with the outcome variable were entered into the multivariable logistic analysis to form the model. Multivariable analysis model using adjusted odds ratio (AOR) was applied to identify the important factors of acceptance on cervical cancer screening and used to control for possible confounding effects. Level of significance below 0.05 was considered to determine the association.

## 3. Results

### 3.1. Sociodemographic Characteristics of the Study Population

From the total 790 samples, 788 women participated in the study with a response rate of 99.7%. The mean age of participants was 30.8 ± 9.2SD years, with a minimum of 18 and a maximum of 70 years. Amhara 785 (99.6%) and Orthodox Christianity followers 756 (95.9%) were the dominant ethnic and religious groups, respectively (Table 1). Three hundred and forty-eight (44.2%) have formal education. More than half of the participants 499 (63.3%) were housewives, and 289 (36.7%) were employed. Four hundred and seventy (59.6%) of them were rural dwellers. Majority of the study participants (558, 70.8%) were economically dependent on their husband or their families.

### 3.2. Knowledge about Cervical Cancer among Women in Dabat District, Northwest Ethiopia, 2017

Among all the study subjects, 366 (46.4%) had heard of cervical cancer. When they were asked about the source of information among those who heard of cervical cancer, health workers were the predominant source (144, 39.3%) followed by media (television and radio) (76, 20.8%) and friends/relatives (74, 20.2%); about 67 (18.3%) heard of cervical cancer from more than one source and 5 (1.4%) from books and pamphlets.

When they were asked about risk factors and symptoms of cervical cancer, 533 (67.6%) of the study participants mentioned at least one risk factor, and 604 (76.6%) identified at least one symptom.

The overall knowledge of cervical cancer was computed by pooling the above three knowledge questions. Based on this information, from the total respondents, only 287 (36.4%) were knowledgeable about cervical cancer.

### 3.3. Perception of Severity towards Cervical Cancer among Women in Dabat District, 2017

Two hundred and sixty (33.0%) of the respondents were worried to develop cervical cancer, and 477 (60.5%) of them thought that all women should undergo cervical cancer screening (perceived severity for cervical cancer).

### 3.4. Awareness of Cervical Cancer Screening among Women in Dabat District, Northwest Ethiopia, 2017 (*n* = 788)

From all the respondents, 185 (23.5%) heard of cervical cancer screening. One hundred and forty-seven (18.7%) respondents knew the availability of screening. From those who knew the availability of cervical cancer screening, 97 (66.0%) of them mentioned hospitals, and 50 (34.0%) mentioned other institutions such as health centers and health posts. From the total respondents, the overall awareness of cervical cancer screening was 95 (12.1%) (95% CI: 9.6, 14.5).

### 3.5. Acceptance of Cervical Cancer Screening among Women in Dabat District, Northwest Ethiopia, 2017

One hundred and thirty-five 17.1% (95% CI: 14.4, 19.8) of them accepted the screening (decided to be screened within the next two months), and 459 (58.2%) of the respondents volunteered for cervical cancer screening (Table 2). Two hundred and eighty-six (36.3%) respondents gave the reason of being healthy for not volunteering for screening.

### 3.6. Factors Associated with Acceptance of Cervical Cancer Screening among Women in Dabat District, Northwest Ethiopia, 2017

A significant difference on the acceptance of cervical cancer screening among respondents in marital status, parity, educational status, resident, occupation, income situation, perceived severity, knowledgeable about cervical cancer, and perceived severity were detected during bivariate logistic regression analysis. But women of multiparous, knowledge about cervical cancer, and perceived the disease as sever were shown to be significant predictors of acceptance for cervical cancer screening when adjusted for variables.

Women who had children 1–4 and 4+ were 1.9 and 3.1 times more likely to accept cervical cancer screening than women who did not give birth (AOR = 1.9, 95% CI: 1.2, 2.9) and AOR = 3.1, 95% CI: 1.7, 5.5), respectively. Knowledgeable women about cervical cancer were 2.6 times more likely to accept screening than women who were not knowledgeable about the disease (AOR = 2.6, 95% CI: 1.7, 3.8), and women perceived as serious of cervical cancer were 1.9 times more likely to accept screening than did not perceive the disease as serious (AOR = 1.9, 95% CI: 1.2, 3.1) (Table 3).

## 4. Discussion

In this study, awareness of cervical cancer screening among respondents was 12.1% which was in agreement with the result in India 16.4% [9] and 18.1% [10], but lower than the study findings from in different parts of Nigeria 34.5% [11] and 25.0% [12]. The difference in the level of awareness may be partly explained by educational status. The highest level of awareness was from studies in urban population, while this study was done in both urban and rural population. Moreover, the study conducted in India, Nigeria, and Ethiopia was facility-based, and women could have access to get information about cervical cancer by health care providers.

Four hundred and fifty-nine (58.2%) of the respondents were willing to undergo cervical cancer screening. This was congruent with the findings in Addis Ababa (62.7%) [15], Nigeria (62.5%) [12], and Uganda (63%) [18]. But it was higher than the report in Addis Ababa 45.4% [16]. The gap might be attributed to the time period of the studies and the change of government policy for chronic diseases particularly cervical cancer screening and prevention recently. In this study, more than half of the respondents volunteered for cervical screening, but only 17.1% of women accepted the screening. This finding was in line with the result in Addis Ababa 24.8% [15]; however, it was lowest from studies conducted in Nigeria (79.8%) and rural Mozambique (86%) [11, 25]. The possible explanation for this variation might be due to the difference in study settings, study participants, and health policy of the countries. The study in Nigeria and Mozambique were health facility-based studies, in which participants might have relatively higher contact with health care providers and access to health information from health professionals.

Regarding factors related to cervical cancer screening acceptance, parity women who had more children were 3 times more likely to accept cervical screening than women who had no child. This finding is contrary to studies conducted in Nigeria and Dares Salaam, Tanzania [11, 22]. The high chance of acceptance for cervical cancer screening among multipara women might be explained by these women who would have experienced repeated exposure in health facilities (during pregnancy, delivery at postnatal period, and other health services) might help them to gain information on sexual and reproductive health issues including the benefits of early cervical cancer screening.

Knowledgeable of women about cervical cancer were 2.5 times more likely to accept cervical cancer screening than women who were not knowledgeable. This finding was parallel to studies in Tunisia, Nigeria, and Addis Ababa, Ethiopia [7, 11, 22], respectively. This is true if a respondent knowing cervical cancer is easily preventable through early screening more likely to accept the screening.

Women who perceived the disease as severe were 2 times more likely to accept cervical cancer screening than women who perceived the disease as not severe. This finding is supported by the study conducted in Kisumu, Kenya [20]. This could be explained by perceived severity leading a woman to be screened earlier before attaining the disease or developing advanced cervical cancer (disseminated to the adjust organs) or resulting in decease. Earlier screening by affordable technology and treatment of precancers are the most promising interventions to reduce the burden of cervical cancer.

### 4.1. Limitation of This Study

Due to the fact that the study was a cross-sectional study describing the cause and effect relationship of the exposure, and outcome was difficult. Practical acceptability was not measured which would have a better estimator of acceptance rate and there might be interviewer bias.

## 5. Conclusion

The study shows that the awareness of cervical cancer and accepting the screening was very low. The finding also revealed that women of multiparous, knowledgeable about cervical cancer and perceived the disease as severe was shown to be significant factors of acceptance for cervical cancer screening.

## Figures and Tables

**Table 1:** Percentage distribution of the study population by selected sociodemographic variables, Dabat, Ethiopia, 2017.

Variables	Frequency	%
*Age*		
<20 years	43	5.5
20–24 years	154	19.5
25–29 years	233	29.6
30–34 years	103	13.0
35–39 years	132	16.8
40+	123	7.5

*Ethnicity*		
Amhara	785	99.6
Tigre	03	0.4

*Religion*		
Orthodox	756	95.9
Muslim	32	4.1

*Marital status*		
Married	557	70.7
Single	123	15.6
Divorced	55	7.0
Widowed	41	5.2
Separated	12	1.5

*Parity*		
Nulliparous	190	24.1
1–4 children	392	49.8
4+	206	26.1

**Table 2:** Cervical cancer screening acceptance among women in Dabat district, 2017.

Variables	Frequency (%)
*Do you intend to screen for cervical cancer?*	
Yes	459 (58.2)
No	329 (41.8)
*If you volunteer, when do you screen for cervical cancer?*	
Within the next two months	135 (17.1)
Within the next six months	119 (15.1)
Within the next year	138 (17.5)
Within the next two years	67 (8.5)

**Table 3:** Factors associated with acceptance of cervical cancer screening among women in Dabat district, Northwest Ethiopia, 2017.

Variables	Acceptance accepted (%)	Not accepted %	COR (95% CI)	AOR (95% CI)
*Marital status*				
Married	88 (15.8)	469 (84.2)	1.3 (0.9–2.0)	0.8 (0.5, 1.3)
Single	47 (25.2)	184 (74.8)	1	1

*Parity*				
Nulliparous	51 (26.8)	139 (73.2)	1	1
Have 1–4 children	64 (16.3)	328 (83.7)	3.4 (1.9–6.0)^*∗*^	**1.9 (1.2, 2.9)** ^*∗*^
Have 4+ children	20 (9.7)	186 (90.3)	1.8 (1.2–2.9)	**3.1 (1.7, 5.5)** ^*∗∗*^

*Educational status*				
Have formal education	82 (23.6)	266 (76.4)	2.3 (1.5–3.3)	1.0 (0.6–1.9)
Have no formal education	53 (12.0)	387 (88.0)	1	1

*Resident*				
Urban	71 (22.3)	247 (77.7)	1.8 (1.3–2.6)^*∗*^	0.8 (0.5–1.2)
Rural	64 (13.6)	406 (86.4)	1	1

*Occupation*				
Housewives	65 (13.0)	434 (87.0)	1	1
Employee and student	70 (24.2)	219 (75.8)	2.1 (1.5–3.1)^*∗∗*^	1.2 (0.6–2.3)

*Income situation*				
Independent	50 (21.7)	180 (78.3)	1.5 (1.0–2.3)	0.9 (0.5–1.6)
Dependent	85 (15.2)	473 (84.8)	1	1

*Knowledge*				
Knowledgeable	79 (27.5%)	208 (72.5)	3.0 (2.0–4.4)^*∗∗*^	**2.6 (1.7, 3.8)** ^*∗∗*^
Not knowledgeable	56 (11.2)	445 (88.8)	1	1

*Perceived severity*				
Perceived as severe	28 (10.8)	232 (89.2)	**2.1 (1.3–3.3)** ^*∗*^	**1.9 (1.2, 3.1)** ^*∗*^
Perceived as not severe	107 (20.3)	421 (79.7)	1	**1**

^*∗*^ *p* value <0.05 and ^*∗∗*^*p* value <0.001. COR = crude odds ratio, OR = adjusted odds ratio.

## Data Availability

The datasets used and/or analyzed during the current study will be available from the corresponding author upon request.

## Abbreviations

ANRS: Amhara National Regional State
CC: Cervical cancer
CCS: Cervical cancer screening
EDHS: Ethiopia Demographic Health Survey
HIV: Human immune deficiency virus
KM: Kilometer
ROCs: Reproductive organ cancers
STIs: Sexually transmitted infections
WHO: World Health Organization.
